# Renal perfusion examination with contrast‐enhanced ultrasound in vascular surgery

**DOI:** 10.1002/agm2.12352

**Published:** 2024-08-19

**Authors:** Zhiyuan Wu, Ning Zhao, Yongjun Li

**Affiliations:** ^1^ Department of Vascular Surgery, Beijing Hospital, National Centre of Gerontology, Institute of Geriatric Medicine Chines Academy of Medical Sciences Beijing China

A recent consensus on the standardized evaluation of renal cortical perfusion with contrast‐enhanced ultrasound in elderly patients in China was published. This paper mainly aimed to briefly introduce renal perfusion examination with CEUS in vascular surgery.

The kidneys, being the most vascularized organ in the human body, are highly susceptible to inadequate blood perfusion. In a resting state, the blood flow through both kidneys in healthy adults is approximately 1200 mL/min, equivalent to 25% of cardiac output.^1^ Approximately 94% of this renal blood flow is directed to the renal cortex.^2^ The kidneys maintain the ability to autonomously regulate blood flow within an arterial blood pressure range of 80–180 mmHg. Renal perfusion pressure typically refers to the difference between renal artery pressure and renal vein pressure. In 1991, Gosling et al.^3^ introduced the concept of the critical closing pressure to reflect the actual renal perfusion.

Renal perfusion can be influenced by various factors including physiological, pathological, pharmacological, traumatic, and surgical factors. Physiological factors primarily involve hemodynamic parameters (blood pressure, blood volume, cardiac output, blood viscosity, etc.) and the sympathetic‐parasympathetic nervous system. Specifically, renal diseases or renal vascular‐related disorders constitute the primary pathological factors affecting renal perfusion. These renal vascular‐related disorders can be categorized into primary and secondary renal vascular lesions. The former encompasses renal artery atherosclerotic stenosis, renal artery dysplasia, Takayasu arteritis, renal artery aneurysm, renal artery thrombosis or embolism, renal artery dissection, renal vein thrombosis, etc. Secondary renal artery lesions may include aortic dissection, para‐renal or supra‐renal abdominal aortic aneurysm,^4^ renal artery metastatic cancer,^5^ etc. Other rare renal vascular diseases such as renal arteriovenous fistula, congenital renal artery anomalies, nutcracker syndrome, can also impact renal perfusion. Among these, renal artery atherosclerosis, renal artery fibromuscular dysplasia, and Takayasu arteritis are currently the three most common causes of renal artery stenosis (RAS). Unlike Western populations, in the Chinese population, Takayasu arteritis is more commonly observed than renal artery fibromuscular dysplasia.

RAS can lead to renal dysfunction and renovascular hypertension. The reduction in renal blood flow caused by RAS results in decreased renal perfusion, triggering a series of pathological changes, including renal ischemia, glomerular atrophy and sclerosis, interstitial fibrosis, and tubular injury, ultimately leading to impaired renal function. RAS induces glomerular atrophy and sclerosis, leading to a decrease in glomerular filtration rate and affecting renal filtration function. Moreover, it causes tubular injury, impairing the absorption and excretion functions of renal tubules, thereby affecting urine formation and excretion. Additionally, RAS leads to hypertension through various mechanisms.^6^ Reduced renal perfusion stimulates the synthesis and release of renin, activating the renin‐angiotensin‐aldosterone system (RAAS), resulting in elevated aldosterone levels, increased blood volume, and consequently elevated blood pressure. RAS‐induced renal ischemia activates the sympathetic nervous system, increasing heart rate and myocardial contractility, constricting peripheral blood vessels, and causing hypertension. Renal ischemia inhibits the generation of vasodilators (such as nitric oxide and prostaglandins), leading to decreased vasodilatory capacity and exacerbated vasoconstriction, thereby precipitating hypertension.

Methods to improve renal perfusion include pharmacological therapy, endovascular treatment (percutaneous transluminal angioplasty or stenting), and open surgical treatment. Vasodilators such as ACE inhibitors and ARBs can enhance renal perfusion by dilating renal blood vessels, reducing renal arteriolar resistance, and improving renal blood flow. Antiplatelet drugs such as aspirin can improve renal microcirculation and increase renal blood flow by inhibiting platelet aggregation. Surgical interventions (endovascular management and open surgical repair) can also improve renal perfusion by relieving RAS and restoring normal blood flow supply to the kidneys. However, multiple guidelines currently suggest that balloon angioplasty or stenting of RAS does not confer significant advantages over pharmacological therapy alone.^7^, ^8^, ^9^ This is attributed to various factors including limitations in common RCT studies such as small sample sizes and high heterogeneity in inclusion and exclusion criteria. Another commonly overlooked issue lies in the assessment of clinical endpoints in observational studies. The endpoints typically include cardiovascular events, as indicated in the CORAL study,^10^ as well as changes in hypertension, related mortality, or primary patency of renal arteries. However, assessment of renal perfusion can also be considered as one of the endpoints.

Detecting changes in renal perfusion is significant for the diagnosis of renovascular diseases, determination of etiology, and estimation of prognosis. Currently, methods for detecting renal perfusion primarily include Color Doppler semi‐quantitative blood flow grading (CDFI), power Doppler ultrasound(PDU), renal artery blood flow waveform, and renal resistance index (RRI),^11^ renal contrast‐enhanced ultrasound (CEUS), near‐infrared spectroscopy technology (NIRS), and blood oxygen level‐dependent magnetic resonance imaging (BOLD MRI), etc. Among these, CDFI can clearly display the blood flow images of various vessels in the kidneys, with arterial and venous blood flow branching from the renal hilum to the cortex, gradually transitioning from coarse to fine. PDU demonstrates superiority over CDFI in assessing renal parenchymal blood flow perfusion, particularly in the evaluation of low‐flow and low‐energy blood flow.^12^ NIRS enables the detection of local tissue oxygenation status by utilizing near infrared light with good tissue penetration, penetrating through the skin into the tissue, and subsequently measuring tissue oxygen saturation.^13^ BOLD MRI can evaluate renal tissue oxygenation status without exposure to radiation or exogenous contrast agents.^14^

Contrast‐enhanced ultrasound (CEUS) involves the injection of microbubbles of contrast agents into the bloodstream, utilizing the unique properties of contrast agents and specific imaging techniques to clearly display microvascular blood flow signals containing the contrast agent. This allows for the reflection of the corresponding microvascular perfusion, making it a novel technology for monitoring organ blood flow perfusion in clinical practice.^15^ In recent years, CEUS technology has rapidly advanced. CEUS for assessing renal perfusion has been widely applied in vascular diseases. It can be used to evaluate the baseline status of renal perfusion, helping vascular surgeons with surgical strategies, such as in cases of Takayasu arteritis, renal atherosclerotic stenosis.^16^, ^17^ Furthermore, compared to biochemical indicators like creatinine, CEUS for assessing renal perfusion can early reflect vascular‐related damage to the kidneys, such as renal injury caused by acute lower limb ischemia, renal hypoperfusion due to false lumen supply to the renal artery in aortic dissection, or hypoperfusion caused by renal artery dissection or embolism. CEUS can also be used to evaluate the impact of vascular surgical procedures on renal perfusion, particularly before and after stent implantation for RAS,^18^ or changes in corresponding renal perfusion when reconstructing renal artery branches in complex aortic diseases,^4^ or the impact of extensive use of contrast agents in CT examination. Assessing surgical outcomes and the recovery of renal function helps physicians adjust postoperative treatment plans promptly, ensuring patients achieve optimal therapeutic effects. Additionally, by evaluating renal perfusion with CEUS, physicians can assess the risk of complications such as postoperative renal insufficiency, renal infarction, or renal vascular thrombosis.

The integration of multiple imaging modalities holds significant promise for enhancing diagnostic accuracy and evaluation of renal diseases. CEUS stands out as a valuable adjunct to traditional imaging techniques such as computed tomography (CT) and magnetic resonance imaging (MRI). However, CT may induce radiation, and both CT and MRI are more expensive and have lower accessibility, CEUS can serve as a first‐line imaging method in some conditions.^19^ By harnessing the complementary strengths of different imaging modalities, a comprehensive understanding of renal pathology can be achieved. Moreover, advancements in CEUS technology, including improvements in microvascular resolution and optimization of contrast agents, coupled with the integration of artificial intelligence,^20^ are poised to further enhance the quality of CEUS imaging and interpretation. As the census stated, the most used microbubble contrast agent is SonoVue, which is metabolized via the lung, making it more friendly to patients with liver or renal insufficiency.

In the future, more large‐scale prospective studies on the renal perfusion assessment with CEUS are required to guide the clinical practice. As shown in endovascular aneurysm repair,^21^, ^22^ artificial intelligence is an optimal candidate tool for these studies or for the analysis software. Furthermore, more CEUS contrast should be developed to meet various needs for the assessment.

In summary, renal perfusion assessment, particularly through CEUS, plays a pivotal role in vascular surgery by aiding in the evaluation of renal blood supply, guiding surgical decision‐making, predicting postoperative complications, and assessing surgical outcomes. These advancements contribute to improving the success rate of surgical interventions and enhancing patient prognosis.

## AUTHOR CONTRIBUTIONS

YL designed the conception and revised the manuscript. ZW and NZ wrote the manuscript. ZW revised the manuscript. All authors reviewed and approved the manuscript.

## CONFLICT OF INTEREST STATEMENT

Yongjun Li, do not have a financial interest/arrangement or affiliation with one or more organizations that could be perceived as a real or apparent conflict of interest in the context of the subject of this manuscript.

## FUNDING INFORMATION

This work was supported by CAMS Innovation Fund for Medical Sciences (CIFMS‐2021‐I2M‐1‐050) and PUMC Discipline Construction Project (No.201920102101).
